# Climate change and mental health: Position paper of the European Psychiatric Association

**DOI:** 10.1192/j.eurpsy.2024.1754

**Published:** 2024-05-23

**Authors:** Lasse Brandt, Kristina Adorjan, Kirsten Catthoor, Eka Chkonia, Peter Falkai, Andrea Fiorillo, Tomasz M. Gondek, Jessica Newberry Le Vay, Martina Rojnic, Andreas Meyer-Lindenberg, Andreas Heinz, Geert Dom, Jurjen J. Luykx

**Affiliations:** 1 Department of Psychiatry and Psychotherapy, Charité – Universitätsmedizin Berlin, Charité Campus Mitte, Corporate Member of Freie Universität Berlin, Humboldt Universität zu Berlin, and Berlin Institute of Health, Berlin, Germany; 2 German Center for Mental Health (DZPG), Germany; 3 Department of Psychiatry and Psychotherapy, School of Medicine, Ludwig-Maximilians-University of Munich, Munich, Germany; 4 University Hospital of Psychiatry and Psychotherapy, University of Bern, Bern, Switzerland; 5 Estates-General of Mental Health, Kortenberg, Belgium; 6 Flemish Association of Psychiatry, Kortenberg, Belgium; 7 Collaborative Antwerp Psychiatric Research Institute (CAPRI), University of Antwerp, Antwerp, Belgium; 8 Ziekenhuis Netwerk Antwerpen, Psychiatrisch Ziekenhuis Stuivenberg, Antwerp, Belgium; 9 Department of Psychiatry, Tbilisi State Medical University, Tbilisi, Georgia; 10 Department of Mental Health, Collaborating Centre for Research and Training, University of Campania “L. Vanvitelli” & WHO, Naples, Italy; 11 Iter Psychology Practices, Wroclaw, Poland; 12 Institute of Global Health Innovation, Faculty of Medicine, Imperial College London, London, UK; 13 Grantham Institute - Climate Change and the Environment, Faculty of Natural Sciences, Imperial College London, London, UK; 14 University Hospital Centre Zagreb, Zagreb, Croatia; 15 School of Medicine, University of Zagreb, Zagreb, Croatia; 16 Central Institute of Mental Health, Department of Psychiatry and Psychotherapy, Medical Faculty Mannheim, Heidelberg University, Mannheim, Germany; 17 Bernstein Center of Computational Neuroscience, Berlin, Germany; 18 Berlin School of Mind and Brain, Berlin, Germany; 19 Faculty of Medicine and Social Sciences, University of Antwerp, Wilrijk, Belgium; 20 Department of Psychiatry and Neuropsychology, School for Mental Health and Neuroscience, Maastricht University Medical Centre, Maastricht, The Netherlands; 21 Department of Psychiatry, Amsterdam Public Health Research Institute, Amsterdam University Medical Center, Vrije Universiteit Amsterdam, Amsterdam, The Netherlands; 22 Outpatient Bipolar Disorders Clinic, GGZ InGeest Mental Healthcare, Amsterdam, The Netherlands

**Keywords:** climate change, European Psychiatric Association, mental health, position, psychiatry

## Abstract

**Background:**

Climate change is one of the greatest threats to health that societies face and can adversely affect mental health. Given the current lack of a European consensus paper on the interplay between climate change and mental health, we signal a need for a pan-European position paper about this topic, written by stakeholders working in mental health care.

**Methods:**

On behalf of the European Psychiatric Association (EPA), we give recommendations to make mental health care, research, and education more sustainable based on a narrative review of the literature.

**Results:**

Examples of sustainable mental healthcare comprise preventive strategies, interdisciplinary collaborations, evidence-based patient care, addressing social determinants of mental health, maintaining health services during extreme weather events, optimising use of resources, and sustainable facility management. In mental health research, sustainable strategies include investigating the impact of climate change on mental health, promoting research on climate change interventions, strengthening the evidence base for mental health-care recommendations, evaluating the allocation of research funding, and establishing evidence-based definitions and clinical approaches for emerging issues such as ‘eco-distress’. Regarding mental health education, planetary health, which refers to human health and how it is intertwined with ecosystems, may be integrated into educational courses.

**Conclusions:**

The EPA is committed to combat climate change as the latter poses a threat to the future of mental health care. The current EPA position paper on climate change and mental health may be of interest to a diverse readership of stakeholders, including clinicians, researchers, educators, patients, and policymakers.

## Introduction

Climate change is among the anthropogenic processes with the most critical impact on the equilibrium of Earth’s systems. The United Nations Framework Convention on Climate Change defines climate change as the change of climate, which is attributed directly or indirectly to human activity that alters the composition of the global atmosphere [1]. Notably, the change in climate is in addition to natural climate variability observed over comparable time periods and caused by human activity [1]. Environmental studies indicate that Earth is now outside of a safe operating space for humanity and that anthropogenic effects such as climate change, loss of biodiversity, and pollution interact and show aggregate effects on Earth’s systems [2]. Carbon dioxide and other greenhouse gas (GHG) emissions, such as methane, contribute significantly to the rising global surface temperatures [3]. According to the United Nations’ Intergovernmental Panel on Climate Change (IPCC), the world is currently experiencing the largest increase in the Earth’s surface temperature in over 2000 years [3]. Heat extremes, that is, temperatures exceeding previous maxima, have been observed in most inhabited regions of the world and there is unequivocal evidence for the human contribution to heat extremes [3]. These heat extremes are associated with risks to physical and mental health [4]. Vulnerable groups, such as young children and older people over the age of 65 years, are particularly affected by the increase in heatwaves [5]. Climate change leads to an increase in extreme weather and disasters and causes worsening of existing inequalities regarding psychosocial and economic factors [5].

Given the importance of climate change for mental health(care) on the one hand and the lack of a European consensus paper on the interplay between climate change and mental health on the other, we signal a need for a pan-European position paper about this topic, written by stakeholders working in mental health care. The EPA is committed to combat climate change as the latter poses a threat to the future of mental health care. Therefore, on behalf of the EPA we give recommendations to make mental health care, research, and education more sustainable. To that end, we start the position paper by summarising the impact of climate change on mental health, then discuss strategies to increase sustainability in mental health care, and end by providing recommendations for people working in mental health patient care, research, and education across Europe.

## Methods

We performed a narrative review of the literature. The databases PubMed, MEDLINE, and Web of Science were searched from database inception up until April 2024, without restrictions to language or country of origin of the study or publication date (search terms: “climate change” AND “mental health”). We manually searched references of the included studies and performed additional selective searches with a search engine (i.e., Google Scholar). We included original research and reviews focussing on climate change and mental health. The articles we retrieved were reviewed and qualitatively synthesised according to the effects of climate change on mental health.

## Impact of climate change on mental health

**Figure 1.**
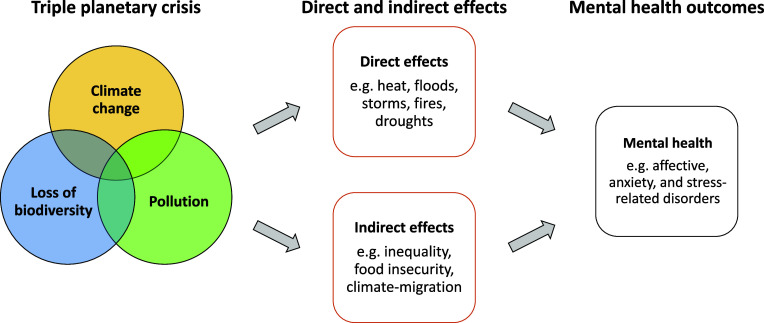
Direct and indirect effects of climate change, loss of biodiversity, and pollution (i.e. triple planetary crisis [9]) on mental health.

The number of scientific articles on the impact of climate change on mental health has increased significantly over the last two decades [5, 6], providing evidence of the negative effects of climate change on mental health [4, 5]. Climate change can have a direct impact on mental health, for example through heat, extreme weather, disasters, and air pollution [7, 8]. Indirect negative impacts include food insecurity, climate-associated migration, and climate inequality (Figure 1). Direct and indirect consequences of climate change are interconnected and all pose threats to mental health, particularly for vulnerable groups with limited coping capacities and pre-existing mental disorders [4].

Climate change affects mental health across borders and there is a need for international psychiatric organisations and representatives to advocate for evidence-based positions and policies regarding climate change and mental health. The EPA aims to address this omission in the literature with the current position paper that includes recommendations for sustainability in mental health care, research, and education. The current position paper by the international task force of the EPA complements other position papers by national organisations such as the Royal College of Psychiatrists and the German Association for Psychiatry, Psychotherapy and Psychosomatics (DGPPN) [7, 10].

In the section below, we discuss the direct and indirect effects of extreme weather events, disasters, increases in ambient temperature, and air pollution on mental health as well as discuss forms of mental distress due to climate change. In keeping with the IPCC, we focus on climatic impact drivers, such as disasters (e.g. flooding) and extreme heat and highlight the available evidence [3]. Climate change is the focus of this position paper, but it is very important to note that climate change is connected to a multitude of other planetary-scale environmental processes and ecological crises, such as loss of biodiversity [2].

### Extreme weather events and disasters

Climate change is leading to an increase in extreme weather and disasters [5]. Extreme weather events include floods, storms, fires, and droughts [5]. Extreme weather events can reach the scale of disasters, threatening physical integrity and destroying livelihoods and critical infrastructure [3, 5].

Extreme weather events and disasters can result in significant mental distress via different pathways [4, 11, 12]. These pathways include the experience of mortal danger, the threat to livelihoods, limited access to health care, and involuntary relocation, as well as the loss of property, work, and social support [4, 11]. The mental symptom severity depends on the extent to which the person is affected by the environmental event [11, 12] and the symptoms can last for years [13]. A recent systematic review of studies from South and Southeast Asia identified risk factors in demographic, economic, health, disaster exposure, psychological, and community factor domains. For example, the following were found to be risk factors for mental health disorders in the recent systematic review: severity of disaster exposure, lower education, and financial stress [14].

The prevalence of post-traumatic stress disorder (PTSD) increases after disasters [15, 16]. After Hurricane Katrina, one in three residents of New Orleans showed symptoms of PTSD [16]. Symptoms of anxiety, psychosis, and depression including suicidal thoughts may increase following extreme weather and disasters [12, 17, 18]. Floods are among the most frequently recorded extreme weather events worldwide [7, 19]. One year after a flood in England, 36% of the regional population suffered from PTSD, around a quarter suffered from anxiety disorders and a fifth from depression [20]. In follow-up studies, the persistence of symptoms in those affected by the floods was demonstrated even after several years [13]. The prevalence of depression and anxiety symptoms was two to five times higher in people who were affected by flooding at home than in people who were not affected by flooding [21]. In another example, it was highlighted that half of the residents of New Orleans suffered from an affective or anxiety disorder in the 30 days following Hurricane Katrina [16].

There is also an increased prevalence of affective disorders following droughts, bushfires, and forest fires [4, 17, 18]. Droughts are increasing in severity and frequency due to climate change [7] and droughts are a major driver of climate-associated migration [22]. As a result of droughts, vulnerable groups, such as women, individuals with low socioeconomic status, minors, and older individuals, are at increased risk of mental health problems [4]. There are also indications of increases in alcohol and other substance use and domestic violence following disasters [4].

### Increases in ambient temperature

Since the end of the nineteenth century, the average global surface temperature has increased according to data since 1850 [23]. The rise in temperature has been especially fast over the past fifty years, with an increase in average global surface temperature of 0.2°C per decade [3, 24]. Compared with the average global surface temperature, Europe is warming even faster [3, 24]. Europe’s land areas were 2.04 to 2.10°C warmer in the past 10 years than during the pre-industrial period [3, 24]. Temperatures are projected to increase further, particularly in north-eastern Europe, northern Scandinavia, and inland areas of Mediterranean countries, while slower increases in temperature are projected for western Europe [3, 24]. The increase in temperature includes average global surface temperature as well as heat extremes. Both aspects are associated with negative mental health outcomes [3].

Heat has emerged as one of the most comprehensively studied aspects of climate change in the context of mental health [5]. In the general population, periods of heat are associated with increases in mental health problems, such as stress and negative emotions [25]. Heat leads to increased mortality [26] and psychiatric disorders are a leading risk factor for heat-related deaths [27, 28]. An increased mortality risk was identified for organic mental illnesses such as dementia [26]. In vulnerable persons, such as persons with mental disorders, the potential impact of medication on regulation of body temperature, fluid balance, and electrolytes should be assessed [28, 29].

A recent meta-analysis reported that at high ambient temperatures an increase in average temperature by 1 degree Celsius is associated with a 0.9% increase in mental health morbidity [26]. Another recent systematic review indicated that individuals with mental disorders were at risk of increased morbidity and mortality compared with individuals without mental disorders over a single day with high temperatures [30]. In addition, both global warming and heat waves are associated with increases in acute admissions to psychiatric clinics and emergency departments [26, 31, 32].

These findings raise the question if involuntary admissions might increase due to climate-associated factors. Studies in one region in Greece and one city in Italy indeed indicate that maximum temperatures are positively associated with involuntary admissions [33, 34]. Unpublished work based on numerous weather stations and thousands of involuntary admissions in the Netherlands indicates that mean average temperature is positively associated with involuntary admissions, with projected increases in involuntary admissions owing to climate change of up to 60 yearly by 2050 (manuscript submitted).

Heat is also associated with more aggression among inpatients [35, 36]. A dose-response relationship was found between increasing heat and increasing aggressive incidents in inpatient settings [36]. Possible reasons for this correlation are insufficient opportunities to lower the temperature in inpatient settings, which may lead to reduced quality of sleep as well as limited opportunities for physical activities during heat, which may increase tension [35, 36].

The effects of heat on suicide rates have also been examined [31, 37, 38]. Using data from several decades for the USA and Mexico, it was shown that suicide rates increased by 0.7% in the USA and 2.1% in Mexico when the average monthly temperature rose by 1°C [37]. The authors predicted, based on a progression of climate change, that 9,000 to 40,000 additional suicides could occur in the United States and Mexico due to temperature increases by 2050 [37].

More research is needed to differentiate between the effects of heat waves and increased average temperatures on mental health as well as investigate potential non-linear relationships between temperature and adverse mental health effects (e.g. an average increase from 20°C to 21°C may be associated with different effects than an increase from 40°C to 41°C).

In summary, these results indicate that heat is a relevant factor for the mental health of individuals with and without pre-existing psychiatric conditions.

### Air pollution

Air pollution includes pollutants such as small matter particles with a diameter of 2.5 microns or less (PM_2.5_) and has been linked to climate change due to fossil fuel use, industrialisation, and urbanisation [5, 7, 10]. Air pollution may have a negative impact on cognitive functioning, including attention, memory, reading comprehension, verbal intelligence, and non-verbal intelligence [39].

In addition, studies suggested an increased risk of mental illness, that is, affective disorders such as depression, with air pollution [40–43]. A recent meta-analysis found that the exposure to air pollutants such as PM_2.5_ and NO_2_ may be associated with the onset of depression [44].

Recent publications discuss neuroinflammatory activation by pollutants as a possible mechanism for the link between air pollution and mental illness [40]. This possible neuroinflammatory mechanism in humans is supported by findings from animal models in which depression-like phenotypes were immunologically induced by pollutants [40, 45]. However, research is needed to better understand the causal links between air pollution and mental illness [46].

Finally, there are interactions between climate change, mental and physical health, and social disadvantage. For example, it has been shown that the influence of local poverty, independently of individual income and educational level, correlates with the extent of mental impairment [47, 48] and that poverty in the neighbourhood is also related to the extent of environmental pollution and reduced green spaces [49].

### Mental distress due to climate change

Climate change can cause individual’s fears about the future, which can be associated with considerable distress [50, 51]. ‘Eco-distress’ refers to negative emotions such as sadness, anger, fear, and hopelessness in relation to climate change and the loss of biodiversity [8, 50]. ‘Climate anxiety’ is a term that partially overlaps in meaning with ‘eco-distress’. ‘Climate anxiety’ refers to a stressful expectation of being affected by climate change and is characterised by pronounced fears [10, 52–54]. In this context, a survey was conducted in 2021 among 10,000 adolescents and young adults aged 16 to 25 years from ten countries [55]. In this survey, 59% of respondents stated that they were extremely or very concerned about climate change. In 45% of individuals, this concern was reported to have an impact on the person’s everyday functioning. These results underline the distress due to climate change in young people. The described forms of mental distress are different from psychological and emotional responses to the climate crisis that should not be pathologised and can be constructive and functional drivers of climate action [56].

The loss of biodiversity in combination with climate change and pollution has been described as the triple planetary crisis and highlights the relevance and interconnectedness of these important issues [9]. A recent systematic review shows that Indigenous Peoples are among the disproportionately affected groups by the negative impacts of loss of biodiversity [57]. The close ties between ecological habitats and Indigenous Peoples’ lived experiences may contribute to the disproportionate negative impact of biodiversity loss on Indigenous Peoples’ well-being [57].

Another term related to the loss of biodiversity is ‘solastalgia’. ‘Solastalgia’ refers to grief concerning the loss of natural habitats, activities, or traditions due to climate change [6]. Human physical and mental health is linked to the state of the natural habitat [7]. Thus, the loss of the natural habitat may negatively impact the mental health of its inhabitants [7]. Indications of ‘solastalgia’ have been detected among youth in Indonesia, Inuit communities in northern Canada, farmers in Australia, communities around the Great Barrier Reef, older individuals in the Torres Strait between Australia and New Guinea, and individuals from Ghana [6, 58]. These findings illustrate the far-reaching consequences and existential threats of climate change and loss of biodiversity.

In the following sections, we will discuss strategies to increase sustainability in mental health care, with the goal of curtailing climate change, which in turn may improve planetary as well as mental health outcomes.

## Towards more sustainable mental health care

Climate change is a challenge for mental health care that needs to be addressed in the key areas of patient care, research, and education [59].

The above-mentioned direct and indirect effects of climate change on mental health may lead to an increased need for mental health care. In particular, mental health-care needs may increase in the areas of stress-related disorders, affective disorders, and anxiety disorders. Care services must adapt to changes in the need for psychiatric and psychotherapeutic treatment. Care approaches should be sustainable and adaptable to meet the potentially increasing and changing needs of populations affected by climatic impact drivers [3].

At the same time, care providers, such as psychiatric and psychotherapeutic institutions, should aim to reduce their own contribution to climate change by increasing the efficiency and resource-conserving processes of their care provision and institutions. Reducing GHG emissions and consumption in care facilities will improve sustainability in mental health care. At the same time, GHG emission reductions alone may not make health care sustainable in the long run, as, for instance, emissions resulting from the use of medication also pose a burden on the environment [7, 10]. Moreover, as mentioned in the introduction, while the scope of the current paper is on climate change, loss of biodiversity should be another important scope of healthcare systems in future endeavours to make healthcare more sustainable [7, 10].

Below, we highlight strategies to increase sustainability in mental healthcare for the key areas of patient care, research, and education.

### Patient care

Clinical processes related to patient care, such as emissions generated by inpatient facilities, contribute to increasing emissions and climate change. In this section, we outline mitigation strategies that aim to reduce emissions and adaption strategies that aim to render mental healthcare more resilient to climate change. Mitigation includes preventive strategies, evidence-based patient care, addressing social determinants, optimising the use of resources, and sustainable facility management measures. Adaption includes interdisciplinary cooperation and maintaining health services during extreme weather events. Of note, important strategies such as preventive strategies, evidence-based patient care, and addressing social determinants are relevant for both mitigation and adaption.

Mitigation in mental health care includes several measures. First, the most sustainable care is the care not needed to be given. Preventive strategies therefore play an important role in sustainable mental health care such as primary prevention of mental disorders and promotion of mental resilience [60]. Approaches that focus on reducing the likelihood of one day needing psychiatric or psychotherapeutic treatment, as well as approaches that address mental health vulnerabilities, are pivotal preventive strategies. For example, mental health services that implement targeted interventions to effectively address the evolving needs of individuals in an early stage could improve the sustainability of the health care system [60]. Furthermore, access to primary care, such as regular consultations with general practitioners, may support physical health as well as mental health [60, 61]. Taken together, a public mental health approach is key to achieve prevention of mental disorders, promotion of mental resilience, and more sustainable healthcare [62].

Second, optimising guideline development processes is advisable (e.g. “living guidelines” that are characterised by frequent guideline updates based on the most current evidence) [63]. A more widespread implementation of “living guidelines” would support up-to-date clinical guidance in line with the rapidly evolving body of evidence in psychiatric research. Evidence-based mental health care based on the most recent data would enable efficient, resource-effective, and sustainable health care.

Third, strategies that promote resilience as well as increased attention to the social determinants of mental health, can reduce the need for inpatient and resource-intensive treatment. Empowerment (e.g. promoting health literacy, self-care, and peer support), access to psychotherapy, online consultations, supporting social networks, reducing poverty, reducing homelessness, reducing social isolation, and promoting employment are considered important steps towards sustainable mental health care [7, 50]. Recent examples from countries such as Australia highlight the need to prepare for increased climate-associated migration and the mental health challenges posed by social and economic adversity [64]. Increased climate-associated migration highlights the importance of culturally sensitive psychiatric and psychotherapeutic interventions and language mediation. Individuals experiencing climate-associated migration may be a vulnerable population in the health care system due to psychosocial stressors before, during, and after migration [46, 65, 66].

Fourth, increasing access to green spaces for the general public as well as mental health institutions may have beneficial effects on well-being and mental health [7]. A recent umbrella review retrieved two meta-analyses examining green spaces and natural environments, detecting associations between increased green spaces and reduction of mental health symptoms, but results were limited due observational designs of a subset of the primary studies [46]. Further research is required to assess the effect of green and blue spaces on the incidence and severity of mental disorders such as affective disorders, anxiety disorders, and stress-related disorders [67, 68].

Fifth, care delivery systems and organisations need to reduce their climate impact. Based on data from the 2022 report of the Lancet Countdown on health and climate change, the GHG emissions per person from the health-care sector ranges between 250 to 1100 kilograms of carbon dioxide equivalent in European countries [5]. In comparison, the USA accounted for more than 1700 kilograms of carbon dioxide equivalent, which is 50 times the emissions of India, but the USA had the sixth lowest healthy life expectancy at birth among the countries in the 2022 report of the Lancet Countdown [5]. These findings illustrate the potential of high-quality health care with lower emissions [5]. Psychiatric hospitals in Europe account for a significant proportion of carbon dioxide emissions per capita, and the inpatient sector is more resource-intensive than the outpatient sector [69]. Strategies to optimise the use of resources in clinical care can include minimising the use of disposable products, using digital interventions in clinical practice, reducing less efficient administrative processes, increasing the proportion of outpatient care, and optimising the use of medications and materials according to guidelines (e.g. examining necessary pharmacological doses [7, 70–74]). It would be important to estimate the effects and potentials of different approaches to monitor, evaluate, and optimise the use of resources in clinical care [7].

Sixth, facility management measures also apply to mental health facilities and include improvements in domains of energy management, mobility, recycling, waste, resource use, food, and procurement [6, 69, 74]. Clinics can adapt organisational structures, such as the introduction of a climate officer and regular resource use analyses. Inclusion of sustainability criteria in clinics’ procurement strategies and public communication strategies (e.g. resource use reports) could support sustainability.

Climate adaptation in mental health care includes several measures. First, in the context of climate change, interdisciplinary cooperation with medical disciplines such as internal medicine and other somatic disciplines is advisable, as climate change affects both physical and mental health. Individuals with pre-existing physical and mental disorders may be particularly vulnerable to the effects of climate change and the deterioration of physical and mental health [4, 5]. Providing optimal medical care for people with mental and physical disorders is part of a sustainable mental health strategy.

Second, mental health services should prepare to deliver their services during extreme weather events and disasters to maintain contact with people who may no longer be able to physically reach mental health providers, for example by providing digital mental health services [7, 50].

In conclusion, both mitigation and adaptation strategies are needed to achieve progress towards more sustainable mental health care.

### Research

In this section, we outline strategies to build understanding of the links between climate change, mental health, and mental healthcare in ways that can inform policy and practice and increase sustainability in mental healthcare research. Specifically, the outlined strategies include establishing evidence-based definitions and clinical approaches for emerging issues such as ‘eco-distress’, investigating the effects of climate change on mental health, promoting research regarding actions on climate change, strengthening the evidence base for policy recommendations, evaluating research funding allocation, and optimising research processes to reduce their emissions.

On a diagnostic level, emerging phenomena described as ‘eco-distress’, ‘solastalgia’, and ‘climate anxiety’ require further research to establish evidence-based definitions (e.g. field trials on diagnostic criteria) allowing for future epidemiological studies on the prevalence and impact. Indeed, clear, evidence-based definitions may help to differentiate between psychological and emotional responses to the climate crisis that are not mental health issues and types of distress that may be a mental health issue. For example, further research is needed regarding the possibility of certain types of eco-distress being a specific phobia [75] while other types of distress are not a mental health issue.

Next, there is a need for psychiatric research to further investigate the effects of climate change and related disasters on (mental) health and broader quality of life. It is important to identify protective and risk factors for environmental effects on mental disorders and quality of life. This may allow the development of targeted preventive strategies and interventions in the context of mental health care and planetary health. Within this context, vulnerable groups, such as individuals with few resources and pre-existing mental disorders, as well as populations affected by climate inequality, e.g., children and adolescents, should be particularly considered. Research is also needed to examine how environmental exposures in relatively poorer neighbourhoods and communities affect physical health and sleep quality, which in turn may affect mental health [49, 76].

On an intervention level, climate mitigation and adaptation actions can simultaneously benefit mental health and mental healthcare [77]. Qualitative and quantitative research is needed to assess the expected benefits of climate action for mental health and mental health care. For example, it is important to further investigate the number of suicides that could be prevented if heat waves became less frequent. It would also be important to assess the economic burden of climate change on the mental health care system (specified for different geographical regions). Specific interventions need to be explored. Within this context, a systematic review has shown benefits of nature-based therapies for mental health outcomes [78]. Possibly, by raising more awareness of such benefits, psychiatric institutions may become more ecologically conscious and will thus be more inclined to promote biodiversity and reduce GHG. Importantly, given the high risks of bias in the included studies [78], we signal a need for high-quality, well-powered randomised-controlled trials examining the potential benefits of nature prescriptions for mental health outcomes.

National and international research funding and policy need to step up on this topic. Mental health research is needed to strengthen the evidence base for policy recommendations to inform how to best prevent and respond to the mental health impacts of climate change. Research funding must be allocated to projects focussing on climate change and mental health to support substantial progress in this urgent area of research. For research collaborations, it is recommended to collaborate globally with researchers and other stakeholders from different regions and different scientific disciplines to improve the relevance and applicability of research.

Finally, in line with improving the sustainability of clinical care processes described above, research processes in themselves should be optimised to reduce their emissions [79–81].

### Teaching

In this section, we outline sustainable aspects in health education. Climate change has a major impact on human physical and mental health, and information on this interaction should be included in the training of health professionals [6, 7]. Planetary health is a relevant concept in the context of climate change since planetary health refers to the health of humans and how it depends on the state of the natural systems [82]. Clinically important aspects of planetary health, including the effects of climate change on mental health, should become a standard part of the curricula of medical programmes at universities. Aspects of planetary health may be integrated with educational courses, ranging from preclinical to clinical courses, which could reflect the interdisciplinary concept of planetary health [82]. Essential information on mental health and climate interactions could be part of advanced medical training, such as specialist training in psychiatry and psychotherapy. For example, training could include the impact of climate change on mental health and its implications for clinical care of the general population and vulnerable groups, both for medical students and psychiatry residents and staff.

Finally, how to reduce the impact of healthcare provision and research itself, should be learned by all health professionals. In that respect, ‘circularity’ strategies (including the steps ‘refuse, rethink, reduce, reuse, repair, refurbish, recycle, and recover’) may constitute a helpful guiding principle to reduce the footprint of one’s own care and care provided by the facility [83].

## Limitations

Our position paper has several limitations. First, this position paper does not include a systematic review of the literature, which limits the comprehensiveness. In this position paper, we aimed to include findings from other systematic reviews and meta-analyses to develop the positions of the EPA based on recent evidence-based literature. Second, this paper communicates the position of the EPA. A future policy paper may be developed together with other stakeholders. Third, connections between climate change and mental health constitute a rapidly evolving field of research with heterogenous study designs, populations, outcome parameters, and preventive and interventional measures. Therefore, several statements and positions put forward by the EPA in the current position paper may not generalise to other geographical regions. Fourth, the focus of this position paper is on climate change. However, other related important environmental issues (e.g. loss of biodiversity) require further comprehensive analysis beyond the scope of this paper.

## Conclusions and recommendations for stakeholders in mental health care, research, and education

The EPA is committed to focus on the important issue of climate change and mental health. In this position paper, we have provided a summary of evidence-based findings regarding the detrimental effects of climate change on mental health. Importantly, based on these findings, we highlighted sustainable recommendations for mental health care, research, and education. This position paper of the EPA includes guidance for a diverse readership of stakeholders in mental health care, research, and education.

The main recommendations are summarised in the table below.

**Table 1:** Table of recommendations

Nr.	Recommendation
1	Preventive and public mental health strategies that focus on reducing the likelihood of needing psychiatric or psychotherapeutic treatment and preventive strategies that address mental health vulnerabilities and inequities are important in promoting sustainability of mental health care. Preventive approaches such as empowerment, health literacy, peer support, and healthy lifestyle factors should be incorporated into mental health strategies
2	Social determinants of mental health, such as social isolation, unemployment, poverty, and homelessness, should be addressed to reduce the need for inpatient and resource–intensive treatment, which significantly contribute to the emissions of the healthcare system.
3	Further research is needed to further investigate the impact of climate change on mental health. Evidence–based mental health strategies and research should consider regional differences in climate change impacts throughout Europe.
4	Targeted mental health strategies are needed to effectively address the evolving needs of individuals in the context of climate change. For example, mental health strategies that include digital approaches could improve access to treatments tailored to individual needs (e.g. availability of both face–to–face and online consultations in case of disasters).
5	Interdisciplinary collaborations with medical disciplines such as internal medicine and other somatic disciplines is advisable as climate change affects both physical and mental health. Umbrella medical organisations such as the European Union of Medical Specialists (UEMS), may facilitate these interdisciplinary collaborations.
6	The clinical relevance of the interactions between climate change and mental health should be included in health education, such as medical student training and specialist training for psychiatrists and psychotherapists.
7	Clinical and consumer (patient, family) stakeholders are encouraged to optimise the use of resources in clinical care, such as minimising the use of disposable products, using digital interventions in clinical practice, reducing less effective processes, increasing the proportion of outpatient care, and optimising the use of drugs and materials according to guidelines. Sustainable facility management measures can include the installation of photovoltaics, the use of contracting and green electricity, and the use of insulation and shading instead of air conditioning. The sustainability of meals and catering in mental health facilities may be increased through ecological and vegetarian options.
8	The ‘greening’ of mental health facilities is recommended and may include boosting biodiversity within the institutional site as well as promoting more research into green prescriptions for mental health outcomes.
9	As an international scientific association, the EPA needs to review its own processes and activities and develop a strategic action plan to improve its organisational sustainability aiming to operate climate neutral within the next decade.
10	We encourage each national society of psychiatry to publish statements and recommendations for more sustainable mental health care, as was recently done in the UK, Germany, and The Netherlands [7, 10, 84].

The above recommendations may hopefully inspire scientific associations, healthcare facilities, and governments to address the interplay between climate change and mental healthcare. Clearly, they are 10 among many other conceivable actions with potential impact to mitigate environmental harm caused by mental healthcare and to adapt to the prognosticated increases in mental distress. Effective, inclusive, and sustainable multilateral actions on individual, social, and political levels are needed to tackle climate change, biodiversity loss, and pollution as promoted by the United Nations Environment Assembly [9]. Moreover, as has been demonstrated by the civil, human, and labour rights movements, societal transformations often start bottom-up. Therefore, in addition to action by governments and national and international societies, we need individuals within the mental health workforce to take local initiatives for a green transformation in mental healthcare to materialize in the years to come.
